# A systems biology investigation of neurodegenerative dementia reveals a pivotal role of autophagy

**DOI:** 10.1186/1752-0509-8-65

**Published:** 2014-06-07

**Authors:** Laura Caberlotto, Thanh-Phuong Nguyen

**Affiliations:** 1 The Microsoft Research, University of Trento Centre for Computational Systems Biology (COSBI), Piazza Manifattura 1, 38068 Rovereto, Italy

**Keywords:** GSK-3β, AMPK, Frontotemporal dementia, Alzheimer’s disease, Lewy bodies disease, Progressive supranuclear palsy, Corticobasal dementia, Pick’s disease, Amyotrophic lateral sclerosis-Parkinsonism/dementia complex

## Abstract

**Background:**

Neurodegenerative dementia comprises chronic and progressive illnesses with major clinical features represented by progressive and permanent loss of cognitive and mental performance, including impairment of memory and brain functions. Many different forms of neurodegenerative dementia exist, but they are all characterized by death of specific subpopulation of neurons and accumulation of proteins in the brain. We incorporated data from OMIM and primary molecular targets of drugs in the different phases of the drug discovery process to try to reveal possible hidden mechanism in neurodegenerative dementia. In the present study, a systems biology approach was used to investigate the molecular connections among seemingly distinct complex diseases with the shared clinical symptoms of dementia that could suggest related disease mechanisms.

**Results:**

Network analysis was applied to characterize an interaction network of disease proteins and drug targets, revealing a major role of metabolism and, predominantly, of autophagy process in dementia and, particularly, in tauopathies. Different phases of the autophagy molecular pathway appear to be implicated in the individual disease pathophysiology and specific drug targets associated to autophagy modulation could be considered for pharmacological intervention. In particular, in view of their centrality and of the direct association to autophagy proteins in the network, PP2A subunits could be suggested as a suitable molecular target for the development of novel drugs.

**Conclusion:**

The present systems biology investigation identifies the autophagy pathway as a central dis-regulated process in neurodegenerative dementia with a prevalent involvement in diseases characterized by tau inclusion and indicates the disease-specific molecules in the pathway that could be considered for therapy.

## Background

Dementia is a clinical syndrome that characterizes many different etiologies, including neurodegenerative, metabolic, vascular, and infectious diseases defined by a cluster of symptoms and signs manifested by difficulties in memory, disturbances in language, psychological and psychiatric alterations, and impairments in daily living activities. The neurodegenerative dementias can be caused by a multiplicity of conditions or diseases that lead to the progressive and irreversible degeneration of specific populations of neurons and their connections. The most common cause of neurodegenerative dementia is Alzheimer's disease, less frequent causes include, among other, Lewy body dementia, frontotemporal dementia, and Prion disease.

Individual neurodegenerative dementia diseases are characterized histologically by varying grades of neuronal loss, gliosis, and abnormal accumulation of proteins. The nature of protein deposition defines the histological classification of each neurodegenerative dementia in two major groups: tauopathies and synucleinopathies, associated with the pathological aggregation of tau or alpha-synuclein proteins in the brain, respectively [1,2]. Despite increasing global prevalence, the precise neurobiological basis and terms for objective diagnosis of neurodegenerative dementias remain controversial, and comprehensive understanding of the neurobiology basis of the diseases remains lacking. Moreover, heterogeneous clinical presentations of the same molecular pathology, comorbidity or unexpected pathologies which characterize the aging brain and a strong clinical and pathological overlap between distinct neuropathological diagnoses render insights in the different disorders extremely important for diagnostic purposes.

The molecular background of the phenotypic variability in neurodegenerative dementia has been investigated and a spectrum of relations between clinical syndromes and molecular features has been identified. Although some proteins have emerged as important players in the mechanism of neurodegeneration, the precise molecular machinery involved in neurodegeneration remains largely unknown.

Systems biology has been paving the way to the exploration of complex associations of diseases and, thus, to the inference of the pathogenic mechanism of a particular disease by considering disease-related components in a large-scale network [3-5]. Although systems biology approach could be limited by its deterministic view of genes as influencing various phenotypes, and by the lack of appreciation of physiological regulation and of cultural and environmental aspects, it could, however, give advantages over the narrow view of what constitutes ‘traditional biology’. Molecular networks, particularly protein-protein interaction networks (PIN), are extraordinarily informative because it is well-known that most cellular components do not solely perform the biological functionality, but interplay with other cellular components in an intricate interaction network [4-8]. Human PIN has been a valuable data resource to study molecular pathogenesis for a wide range of diseases [6-13]. Among those, numerous studies have been carried out to deeply understand the molecular networks related to neurodegenerative diseases (NDs), proposing different methodological approaches including network analysis to study Alzheimer’s disease based on PIN and data integration [14], inference of overlapping regulators of NDs in different organisms [15], pathway-based method to uncover the direct commonality among NDs [16], or reconstruction of NDs network based on PPI networks, regulatory networks, and Boolean networks [17]. In addition to disease genes study, systems biology approach has been also applied to drug target elucidation [18]. Yu et al. proposed a systematic approach that used of Random Forest and Support Vector Machine for predicting drug targets by combining the chemical, genomic, and pharmacological information [19]. In Emig et al. [20], a disease gene expression signature and a high-quality interaction network were integrated using network-based approach to prioritize the list of drug targets. Thus, systems biology application in pharmacology hold promises for drug discovery.

The aim of the present study was to identify key molecular hubs relevant for neurodegenerative dementia using a network-based approach in a context of protein-protein interaction. The diseases studied were: Frontotemporal dementia (FTD), Alzheimer disease (AD), Lewy bodies disease (LBD), Progressive supranuclear palsy (PSP), Corticobasal dementia (CBD), Pick’s disease, Prion disease, Hungtington’s disease and Amyotrophic lateral sclerosis-Parkinsonism/dementia complex. Both Tauopathies and synucleinopathies were included to try to uncover any molecular alteration characterizing these subgroups of dementia-related diseases. This is the first attempt of application of systems biology methodology to reveal the molecular complexity of this subgroup of neurodegenerative disorders integrating not only the current knowledge on the specific diseases (OMIM), but also the drug targets, representing the broadest coverage of the genes that has been considered relevant for the treatment of dementia-associated symptoms. This integration of network analysis in biomedical research has uncovered hidden molecular pathways that are mutual between distinct diseases sharing the common symptoms of dementia and provided further support to the hypothesis of alteration in autophagy as the molecular basis of these groups of neurodegenerative disorders, particularly tauopathies.

## Methods

**Figure 1:**
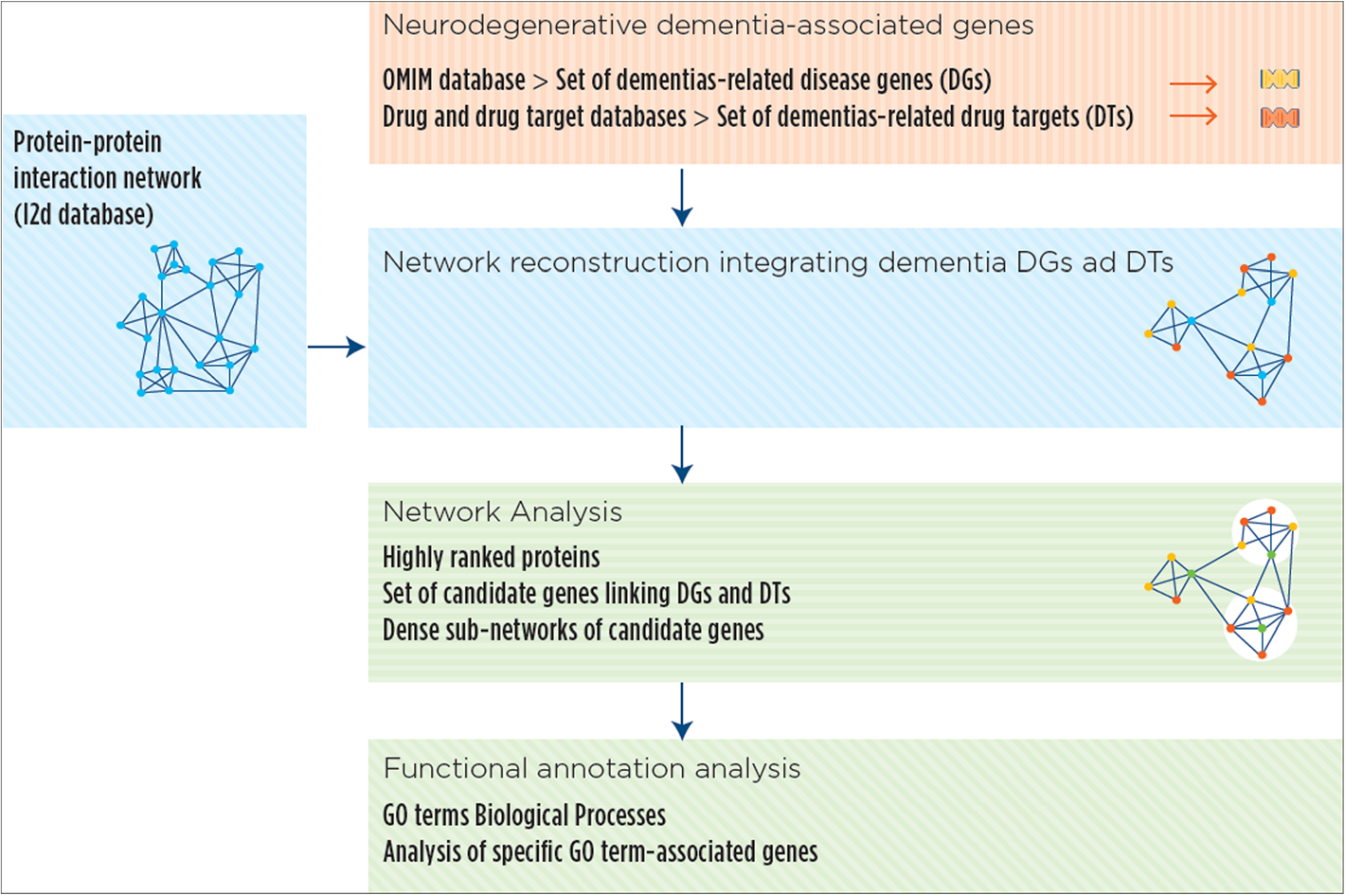
**Schematic representation of the network analysis workflow.** Seed genes provide information on neurodegenerative dementia diseases-associated genes in the OMIM database and on primary molecular drug targets in the different phases of the drug discovery process. Following reconstruction of the protein-protein interaction (PPI) network derived from the integration of seed genes (disease genes and drug targets) and I2D database, we characterized the functional enrichment of the protein in the network by testing over-represented Gene Ontology biological process terms.

The study pipeline is presented in Figure 1 and it is composed by three major steps: (1) Reconstruction of the interaction network by integrating OMIM disease genes, drug target genes and PIN, (2) Analysis of the interaction network of disease genes and drug targets, and (3) Functional annotations analysis of disease genes and drug targets. The three steps are described in details in the following subsections.

### Reconstructing integrating networks of disease genes and drug targets

Three datasets were used to construct the network: OMIM disease genes, drug target genes and protein interaction network.

#### OMIM disease *seed genes*

Disease genes and genetic phenotypes of the different neurodegenerative dementia diseases (Table 1) were extracted from the OMIM database [21], a comprehensive, authoritative compendium of human genes and genetic phenotypes [22]. Keywords that are most relevant for the disorders were defined, such as the official disease names and alternative names. A text mining process was performed to extract genes related to the dementia keywords in the OMIM database.

**Table 1:** Neurodegenerative dementia diseases with their relative disease proteins and protein marker

**Disease**	**Disease protein**	**Official gene symbol**	**Protein marker**
Alzheimer	P05067	APP	Tau
Alzheimer	P01023	A2M	Tau
Alzheimer	P49768	PSEN1	Tau
Alzheimer	Q6ZW49	PAXIP1	Tau
Alzheimer	P49810	PSEN2	Tau
Alzheimer	P29474	NOS3	Tau
Alzheimer	P05164	MPO	Tau
Alzheimer	Q92870	APBB2	Tau
Alzheimer	P02649	APOE	Tau
Alzheimer	P00749	PLAU	Tau
Alzheimer	Q30201	HFE	Tau
Alzheimer	Q92673	SORL1	Tau
Alzheimer	P12821	ACE	Tau
Alzheimer	Q13867	BLMH	Tau
Amyotrophic lateral sclerosis-Parkinsonism/Dementia complex	Q99497	PARK7	Tau
Dementia, familial, non-specific	Q9UQN3	CHMP2B	Tau
Dystonia-Parkinsonism	P21675	TAF1	Tau
Frontotemporal Dementia	Q13148	TARDBP	Tau
Frontotemporal Dementia	P10636	MAPT	Tau
Frontotemporal Dementia	P28799	GRN	Tau
Supranuclear palsy	P10636	MAPT	Tau
Prion	P04156	PRNP	Prion
Prion	P54259	ATN1	Prion
Prion	Q99574	SERPINI1	Prion
Prion	P01920	HLA-DQB1	Prion
Prion, Huntington disease-like 1	P04156	PRNP	Prion/Hungtintin
Huntington Disease	P42858	HTT	Hungtintin
Huntington disease-like 1	P04156	PRNP	Hungtintin
Huntington disease-like 2	Q8WXH2	JPH3	Hungtintin
Huntington disease-like-4	P20226	TPB	Hungtintin
Dementia, Lewy body	P37840	SNCA	Alpha-synuclein
Dementia, Lewy body	Q16143	SNCB	Alpha-synuclein

List of neurodegenerative dementia disease, proteins (Uniprot ID) associated to the disease as obtained from OMIM database and relative Official Gene Symbol and protein markers related to the diseases.

#### Drug target *seed genes*

Drug molecular targets were obtained by collecting information from different pharmaceutical company websites, from a clinical trial database (http://www.clinicaltrial.gov) and from the Drug Bank (http://www.drugbank.ca) (Additional file 1: Table S1). Drugs for the treatment of dementia in all phases of the drug discovery process, from preclinical to marketed drugs, were included. This approach allowed obtaining the broadest coverage of the genes of interest for pharmaceutical drug development to identify the overall key molecular targets of interest for the treatment of dementia. Only primary targets were considered as *seed genes* for network analysis.

#### Protein-protein interaction network

**Figure 2:**
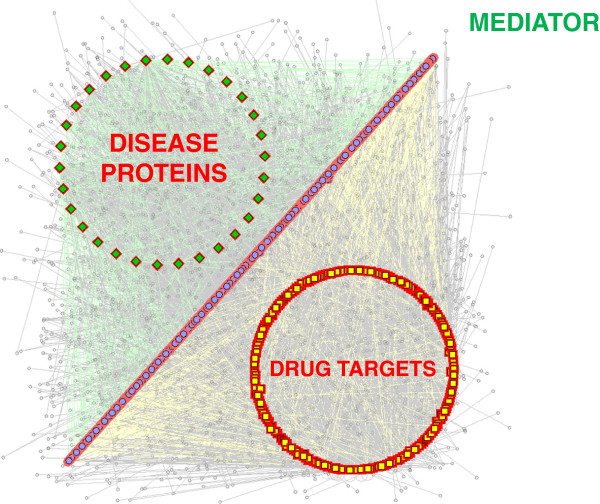
Overview of the PPI network created using disease and drug targets seed proteins and showing their common/shared direct interacting proteins (mediators).

The protein interaction network integrating dementia genes and drug target was extracted from the Interologous Interaction Database (i2d) [23], which is an integrated database of the majority of all known experimental and predicted human protein interaction data sets (including HRPD, BIND, BioGrid, etc.). The database consisted of 846,116 interactions in total, with 173,338 homo sapiens-related. To construct a PIN related to dementia, we firstly extracted the corresponding product proteins of the seed genes (diseases and drug target). We used the ID mapping scheme provided by the Uniprot database to map the seed gene symbols to the Uniprot protein accessions. Consequently, two sets of proteins were obtained, the set of disease proteins *S*_*DG*_ corresponding to the OMIM disease seed genes_*,*_ and the set of drug target proteins *S*_*DT*_ corresponding to drug target seed genes_*.*_ Based on these two sets *S*_*DG*_ and *S*_*DT*_, we then extracted PIN by processing raw data of protein-protein interactions (PPI) in the i2d database. All the homologous predicted protein interactions in the i2d database were excluded to increase the reliability of the protein interaction data. The final interaction network contained the nodes representing disease proteins and drug targets, and the edges representing their protein interactions (Figure 2). We took into account only direct interactions (i.e., one-step neighbors). The network was undirected and unweighted because we considered the binary interactions.

### Network analysis

To gain information on the network and their participating proteins, we evaluated the centrality of proteins in the network. In view of the fact that the functional importance of proteins might be inferred from their central roles in the network [24-26], we computed the degree index for each protein, one of the most applied indices to evaluate the centrality in the network.

A graph *G(E,V)* consists of a set of vertices *(V)* and a set of edges *(E)* between them. An edge *e*_*ij*_ connects vertex *v*_*i*_ with vertex *v*_*j*_. Here, an undirected graph is investigated since our studied protein interaction networks are undirected. An undirected graph has the property that *e*_*ij*_ and *e*_*ji*_ are considered identical. Therefore, the neighbourhood *N*_*i*_ for a vertex *v*_*i*_ is defined as its direct connected neighbours by Equation (1):

(1) $$N_{i}=\left\{v_{j}:e_{\mathrm{ij}}\in E\right\}$$

The degree *D*_*i*_ of a vertex is defined as the number of vertices |*N*_*i*_|, in its neighbourhood *N*_*i*_.

We then computed different network measures to comprehend the topological properties of the constructed network (Table 2).

**Table 2:** Network measures calculated for the integrated network

**Statistics measures**	**Value**
Number of connected components	6
Network diameter	9
Network radius	1
Average shortest path length	3.851
Average number of neighbors	4.209
Network density	0.001
Network centralization	0.120
Network heterogeneity	3.897
Clustering coefficients	0.116

Network measures show the topological properties of the network with different criteria. The first column is the type of measures and the second column is the corresponding values.

• Number of connected components: A connected component is a group of all nodes that are pairwise connected. The number of connected components indicates the connectivity of a network – a lower number of connected components suggest a stronger connectivity.

• Measures to shortest paths: The length of a path is the number of edges forming it. There may be multiple paths connecting two given nodes. The shortest path length, also called distance, between two nodes *n* and *m* is denoted by *L (n,m)*.

• Network diameter: the largest distance between two nodes. If a network is disconnected, its diameter is the maximum of all diameters of its connected components.

• Network radius: the smallest distance between two nodes

• Average shortest path length: also known as the characteristic path length, gives the expected distance between two connected nodes

• Average number of neighbors: indicates the average connectivity of a node in the network

• Network density: a normalized value of the average number of neighbors

• Network centralization: a simple and widely used index of the connectivity distribution. Networks whose topologies resemble a star have a centralization close to 1, whereas decentralized networks are characterized by having a centralization close to 0

• Network heterogeneity: reflects the tendency of a network to contain hub nodes

• Clustering coefficients: In undirected networks, the clustering coefficient *C*_*n*_ of a node n is defined as *C*_*n*_*= 2e*_*n*_*/(k*_*n*_*(k*_*n*_*-1)),* where *k*_*n*_ is the number of neighbors of *n* and en is the number of connected pairs between all neighbors of n. The clustering coefficient is a ratio *N / M*, where *N* is the number of edges between the neighbors of *n*, and *M* is the maximum number of edges that could possibly exist between the neighbors of *n*. The network clustering coefficient is the average of the clustering coefficients for all nodes in the network.

To investigate the proteins potentially related to dementias, we determined mediator proteins, which are defined as proteins that have direct interactions with both proteins in the set of *S*_*DG*_ and of *S*_*DT*_. First, based on the PIN extracted, we searched the direct neighbours *v*_*j*_ of all proteins *v*_*i*_ where *v*_*j*_ ∈ *S*_*DG*_*,* denoted *I*_*DG*_ = {*v*_*j*_}. Similarly, we obtained the set *I*_*DT*_ = {*v*_*k*_}, where *v*_*k*_ is direct neighbours of proteins *v*_*i*_ and *v*_*i*_ ∈ *S*_*DT*_. Then the set of mediator proteins is the intersection set of the two sets *I*_*DG*_ and *I*_*DT*_, denoted *M* by Equation (2).

(2) $$M=I_{\mathrm{DG}}\cap I_{\mathrm{DT}}$$

List of mediator proteins is shown in Additional file 1: Table S1.

### Functional annotation analysis

**Figure 3:**
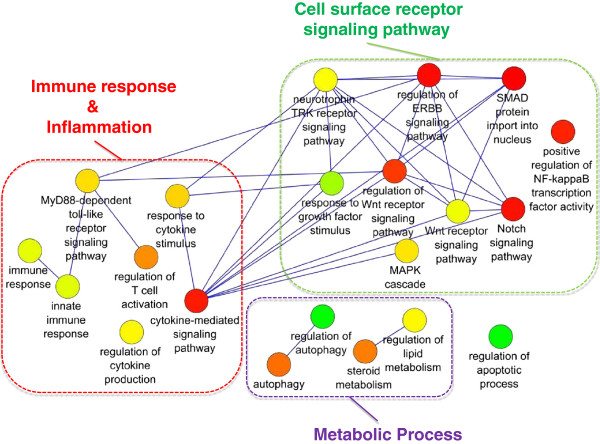
**Summary of statistically significant Gene Ontology biological processes functional annotation corresponding to proteins in the mediator list as obtained from REVIGO **[28]**.** Nodes are GO terms and edges represent the strongest GO terms pairwise similarity. Colors represent the p-values (low values in green, high in red). Only significant GO terms are shown (P < 0.001).

**Figure 4:**
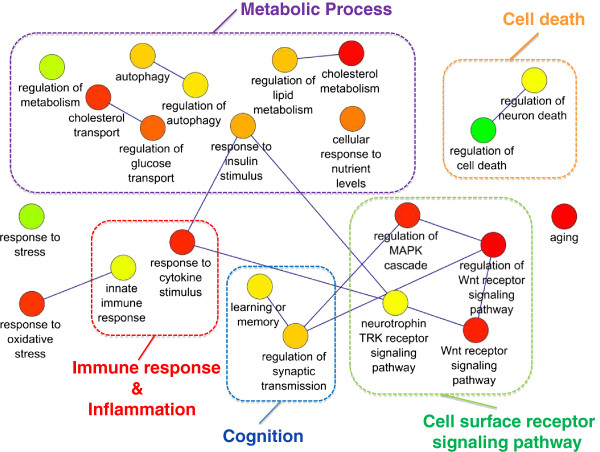
**Representation of statistically significant Gene Ontology biological processes functional annotation corresponding to proteins in the mediator list corresponding to neurodegenerative dementia diseases characterized by tau inclusions as obtained from REVIGO **[28]**.** Nodes are GO terms and edges represent the strongest GO terms pairwise similarity. Colors represent the p-values (low values in green, high in red). Only significant GO terms are shown (P < 0.001).

The complete lists of mediator proteins and the sub-list related to tauopathies (Table 1) were used to extract the most representative GO biological process terms (i.e., the ones that are over-represented, but that do not refer to most general biological processes). For identifying and visualizing enriched GO terms, we used GOrilla and REVIGO tools [27,28]. Hypergeometric distribution was applied to test GO term enrichment, and a p-value threshold of 0.001 was selected. The output graphs were obtained from REVIGO, a web server that considers long lists of Gene Ontology terms and summarizes them by removing redundant GO terms. These terms can be visualized in semantic similarity-based scatterplots and this graph-based visualization accurately renders the subdivisions and the semantic relationships in the data. Each of the GO terms is a node in the graph, and 3% of the strongest GO term pairwise similarities are designated as edges in the graph (Figures 3, 4, and Additional file 2: Figure S2).

In depth analysis of specific GO terms-associated genes was performed. In particular, among the metabolic-related GO terms indicated by the functional enrichment analysis of the complete list of mediators and of the tauopathies-associated sub-list (Figures 3 and 4), autophagy was selected for further analysis. Thus, the proteins list related to GO terms associated to autophagy (GO:0010506 and GO:0006914; Additional file 1: Table S1) were studied and, in addition, for a complete coverage of the autophagy associated genes in the mediator list, other mediator proteins that have been demonstrated to be involved in autophagy process as described in Uniprot (keyword: autophagy) were included in the analysis. In addition to the GO-enrichment analysis, we explored the human autophagy network [29] to investigate the centrality of our mediators in the context of an experimentally validated human autophagy network [29]. Behrends et al. used a modified version of the Comparative Proteomics Analysis Software Suite to identify the autophagy interaction network (AIN) of 409 non-redundant high-confidence candidate interaction proteins (HCIPs), making 751 interactions. They then employed hierarchical clustering in AIN to model ten functional sub-networks. We carried out two different analyses, first on the complete AIN and, second, on the functional clustered network. Firstly we computed the intersection of the mediator list obtained by our method and the list of interacting proteins in the complete AIN, to study the coverage and the topological roles of the mediators. The degree centrality and articulation position were calculated for all mediators based on the AIN. A node is considered an articulation point (or cut vertex) if, and only if, by removing it (and edges through it) we disconnect the graph. Subsequently, to discover the functional roles of mediators related to autophagy, we compared the mediator list with the list of 32 primary baits and 33 secondary baits in the functional sub-networks described in Behrends et al. paper [29].

## Results

We obtained the integrated network consisting of 3,450 proteins and 7,367 interactions. Table 2 shows the statistics of the integrated network. There are 6 connected components and, among them, there exists a giant component (the largest connected components) consisting of 3,435 proteins (99.57% of the total number of proteins) and 7,251 interactions (98.43% of the total number of interactions). Thus, the network is well-connected and comprehensive for network analysis. The shortest path length and neighborhood measures showed that the network is centralized in a number of hubs, and proteins in the network are close to each other and easily reached through short paths. Using the degree index, highly-ranked proteins were extracted as shown in Table 3. The functional annotation analysis of the highly ranked proteins revealed a predominant role of metabolic processes including regulation of energy homeostasis, glucose and lipid metabolism (Additional file 2: Figure S2 and Additional file 1: Table S1). The proteins associated to these metabolic-related GO terms are mainly AMPK subunits (PRKAA1 and PRKAA2) and NF-kB. Cell receptor signaling pathways with terms associate to TRK receptor and Wnt receptor pathways were also significantly enriched (Additional file 1: Table S1).

**Table 3:** List of high-ranked proteins in the dementia network using the degree index

**Uniprot ID**	**Official gene symbol**	**Gene name**	**Degree index**
Q13131	PRKAA1	Protein kinase, AMP-activated, alpha 1 catalytic subunit	419
P54646	PRKAA2	Protein kinase, AMP-activated, alpha 2 catalytic subunit	392
P03372	ESR1	Estrogen receptor 1	250
P19838	NFKB1	Nuclear factor of kappa light polypeptide gene enhancer in B-cells 1	247
Q13547	HDAC1	Histone deacetylase 1	218
Q00005	PPP2R2B	Protein phosphatase 2 (formerly 2A), regulatory subunit B, beta isoform	201
P17252	PRKCA	Protein kinase C, alpha	194
P49841	GSK3B	Glycogen synthase kinase 3 beta	170
P06493	CDK11	Cell division cycle 2, G1 to S and G2 to M	163
P04150	NR3C1	Nuclear receptor subfamily 3, group C, member 1 (glucocorticoid receptor)	156
Q92769	HDAC2	Histone deacetylase 2	138
**P05067**	**APP**	**Amyloid beta (A4) precursor protein**	**136**
P19438	TNFRSF1A	Tumor necrosis factor receptor superfamily, member 1A	133
P67775	PPP2CA	Protein phosphatase 2 (formerly 2A), catalytic subunit, alpha isoform	127
P20226	TBP	TATA box binding protein	126
P24941	Cdk2	Cyclin-dependent kinase 2	125
P30153	PPP2R1A	Protein phosphatase 2 (formerly 2A), regulatory subunit A, alpha isoform	121
P54259	ATN1	Atrophin 1	109
P50750	CDK9	Cyclin-dependent kinase 9	102

In bold is the mediator which is also disease proteins, having direct interactions with drug targets and other disease genes.

Functional enrichment analyses of GO biological process terms were also performed for the complete list of mediators, showing metabolic related terms, immune response and inflammatory processes, and cell surface receptor signaling pathways (Figure 3 and Additional file 1: Table S1). Considering mediator protein associated to Tauopathies, similar functional annotations were presented with the addition of terms related to cell death and cognition-related terms, such as synaptic transmission and learning and memory (Figure 4 and Additional file 1: Table S1). Based on the increasing interest of the role of metabolism in dementia, a major focus was dedicated to the metabolic processes associated terms, in particular to autophagy. As described in Table 4, 27 mediators are involved in autophagy processes or in the regulation of autophagy.

**Table 4:** Autophagy-related proteins and association with dementia disease proteins

**Disease**	**Disease protein**	**Disease protein**	**Mediator autophagy**	**Mediator autophagy**
	**Uniprot ID**	**Official gene symbol**	**Uniprot ID**	**Official gene symbol**
**AD**	**P02649**	**APOE**	**P10636**	**MAPT**
**AD**	P05067	APP	P00519	ABL1
**AD**	**P05067**	**APP**	**P49768**	**PSEN1**
**AD**	**P05067**	**APP**	**P10636**	**MAPT**
**AD**	P05067	APP	P07339	CTSD
**AD**	Q13867	BLMH	Q13131	PRKAA1
**AD**	Q13867	BLMH	Q9BXW4	MAP1LC3C
**AD**	P29474	NOS3	P31749	AKT1
**AD**	**P49810**	**PSEN2**	**P49768**	**PSEN1**
**AD**	Q92673	SORL1	Q9H1Y0	ATG5
**AD**	Q92673	SORL1	Q9C0C7	AMBRA1
**AD**	Q92673	SORL1	Q13131	PRKAA1
**FTD**	Q13148	TARDBP	Q6ZNE5	ATG14/KIAA0831
**FTD**	Q13148	TARDBP	O95166	GABARAP
**FTD**	Q13148	TARDBP	P54646	PRKAA2
**FTD**	Q13148	TARDBP	Q13286	CLN3
**FTD**	Q13148	TARDBP	Q5MNZ9	WIPI1
**FTD**	Q13148	TARDBP	Q676U5	ATG16L1
**FTD**	Q13148	TARDBP	Q9BSB4	C12orf44/ATG101
**FTD**	Q13148	TARDBP	Q9H1Y0	ATG5
**FTD**	Q13148	TARDBP	Q9Y484	WDR45
**FTD**	Q13148	TARDBP	Q9Y4P8	WIPI2
**FTD**	P49768	PSEN1	P10415	BCL2
**FTD**	P49768	PSEN1	Q07817	BCL2L1
**FTD/PSNP**	P10636	MAPT	O60260	PARK2
**FTD/PSNP**	P10636	MAPT	P00519	ABL1
**FTD/PSNP**	P10636	MAPT	P31749	AKT1
**FTD/PSNP**	**P10636**	**MAPT**	**P49768**	**PSEN1**
**FTD/PSNP**	P10636	MAPT	Q13501	SQSTM1
**ALSPD**	Q99497	PARK7	Q6ZNE5	ATG14/ KIAA0831
**ALSPD**	Q99497	PARK7	Q9Y484	WDR45
**ALSPD**	Q99497	PARK7	Q13286	CLN3
**ALSPD**	Q99497	PARK7	Q9H1Y0	ATG5
**ALSPD**	Q99497	PARK7	Q9Y4P8	WIPI2
**ALSPD**	Q99497	PARK7	Q15831	STK11
**ALSPD**	Q99497	PARK7	Q9P2Y5	UVRAG
**ALSPD**	Q99497	PARK7	Q7Z6L1	TECPR1
**LBD**	P37840	SNCA	Q9H0R8	GABARAPL1,3/ATG8
**LBD**	P37840	SNCA	Q92934	BAD

In bold are the mediators protein which are also disease proteins.

**Figure 5:**
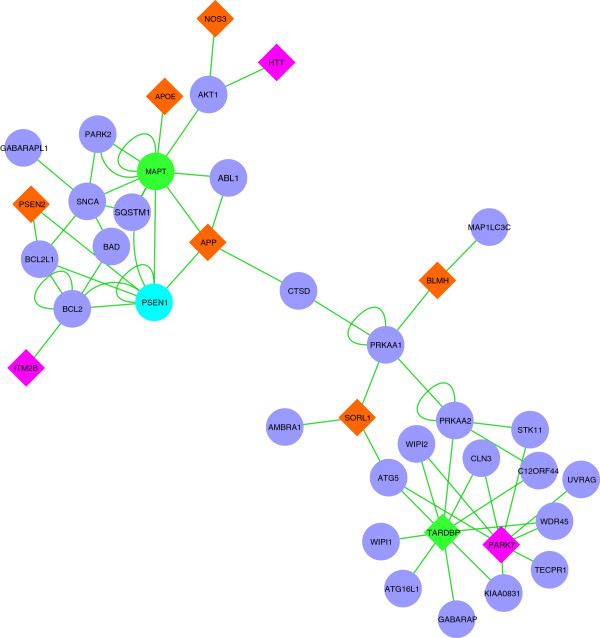
**Neurodegenerative dementia autophagy-associated sub-network.** In the network are indicated the disease proteins (different color for the specific diseases) and the mediator proteins associated. Diamond symbols indicate disease proteins, green is related to FTD, orange to AD, pink to ALSPD, and cyan to proteins that are both AD and FTD (see Table 4). Blue circles represent mediator protein associated to autophagy, colored circles are the protein which are both disease and mediator proteins.

**Figure 6:**
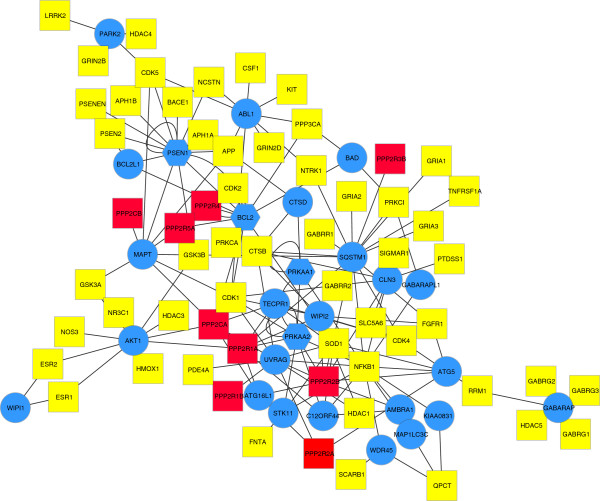
**Autophagy-associated drug target sub-network.** In the network are shown the drug targets (yellow squares) and the associated autophagy-related mediator proteins (blue circles). In red are highlighted the PP2A proteins subunits.

The disease genes associated to the autophagy mediators are shown in Figure 5 and listed in Table 4, while in Figure 6, the drug targets directly associated to the autophagy mediators are represented. A prevalence of subunits of Protein Phosphatase 2A (PP2A), was evident and highlighted in the figure.

**Figure 7:**
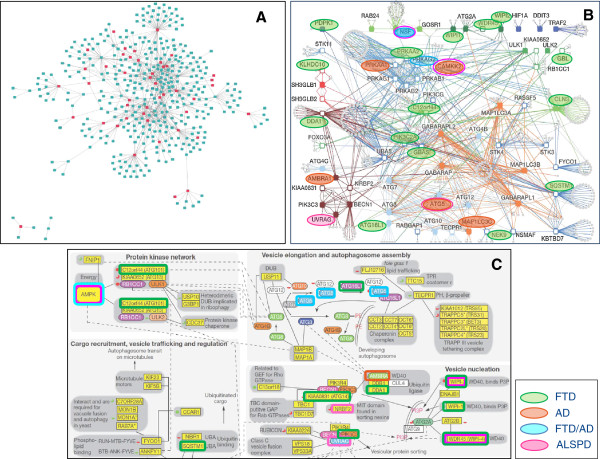
**Integration of neurodegenerative dementia mediator proteins with human autophagy network (modified from **[29]**).** In **A)** mediator proteins of the dementia network are charted in the human autophagy interaction network, in **B)** mediator proteins are mapped in the human autophagy sub-networks and in **C)** mediator proteins are highlighted in the functional integration of the autophagy interaction network.

By investigating the overlaps between the mediator list obtained and the human autophagy network as described in Behrends et al. paper [29], 45 mediators were found in the network (Additional file 3: Table S3). Eight mediators were found in top 10 central nodes in the network by ranking the degree centrality. Moreover, 24/45 mediators were the articulation nodes that are of high interest as the important nodes to prevent network fragmentation (Additional file 3: Table S3). The centrality and crucial positions in the autophagy interaction network of the mediators highlighted their relevant role in the autophagy process (Figure 7A). By analyzing the 10 functional network clustered in the AIN, we found 25 mediator proteins in the network. The autophagy-related mediator proteins were not predominantly belonging to any of the sub-networks described by Behrends and collaborators, but they are present in almost all sub-networks (Figure 7B). FTD-associated mediator proteins (Table 4) were found in protein kinase network, vesicle elongation and autophagosome assembly and vesicle nucleation autophagy phases, while AD-associated proteins were seen only in the protein kinase network, vesicle elongation and autophagosome assembly stages (Figure 7C).

## Discussion

In the present study, network analysis was used to explore from the systems biology perspective, the molecular connections among multifactorial complex diseases with the shared clinical symptoms of dementia, which could suggest related disease mechanisms. A number of diseases were considered, both common (e.g. Alzheimer’s disease) and rare disorders (e.g. amyotropic lateral sclerosis with parkinsonism and dementia) that have as a common and major symptom a progressive and permanent loss of cognitive and mental performance (Table 1).

While previous systems biology studies on disease focus on the disease gene network or drug target network, separately, the method proposed in the current study presented an integrated methodology that can take advantage of both these data, providing further insight into the interactome related to dementia.

Among the most connected proteins (with more than 100 interactions in the network; Table 3) the first 2 proteins in the list were PRKAA1 and PRKAA2, subunits of AMP-regulated kinase (AMPK). AMPK is a central regulator of energy homeostasis controlling neuronal maintenance in response to metabolic stress. Latest research support an involvement of AMPK in Alzheimer [30,31] and, in our previous study on Alzheimer’s disease, on a separate set of data and with a very different systems biology methodological approach, AMPK-related genes were also found to be strongly associated to the disease [14]. Moreover, abnormal neuronal accumulation of activated AMPK (pAMPK) has been described in different tauopathies including PSP, AD, Pick’s disease, and CBD [32]. Thus, the present findings support once more the proposed hypothesis of an alteration of metabolic functions and energy regulation in dementia.

Considering the complete list of mediator proteins, Gene ontology (GO) enrichment analysis confirmed a significant involvement of metabolic signaling regulating energy homeostasis, lipid and glucose metabolism (Figure 3). Metabolic disturbances have been strongly associated to or considered a predisposing factor in AD and a metabolic/signal transduction hypothesis for AD and other tauopathies has been suggested by Iqbal et al. [33]. Amongst the metabolic-related terms, a role for autophagy and regulation of autophagy was highlighted (Figures 3 and 4). Although autophagy has been known for decades, its relevance in neurons and glial physiology has been demonstrated only recently [34]. Autophagy is involved in the intracellular turnover of proteins and cell organelles [35,36] and AMPK is one of its main regulator [37]. In neurons, it is involved in cellular homeostasis and cellular protein clearance pathway and for the remodeling of terminals in support of neuronal plasticity [38]. In glial cells, autophagy is implicated in the elimination not only of glial proteins, but also of those secreted by neurons, which otherwise would accumulate in the extra-neuronal space [39], and it is activated in astrocytes following injury [40]. Thus, it is not surprising that neurodegenerative dementia diseases, which have been linked to the abnormal accumulation of proteins and to alteration of synaptic plasticity, have been associated to the autophagic system [41]. Moreover, a potential role of autophagy in dementia is also suggested by the expression profile extracted from Mantra (http://mantra.tigem.it/), a transcriptional response database of FDA approved drugs, of 2 drugs clinically in use for the treatment of Alzheimer’s disease: galantamine and memantine. Several genes are modulated including AMBRA1, GABARAPL1, CLN3, SQSTM1, and AMPK subunits.

Detailed examination of autophagy-related genes in the mediator list, showed a preferential association to tauopathies, as demonstrated also by the GO enrichment study in the subset of mediators linked to dementia disease characterized by Tau protein inclusions (Figure 4). Autophagy process consists of several sequential steps including protein kinase network regulating the system, vesicle elongation, autophagosome assembly, and vesicle nucleation (Figure 7C) [29] and specific autophagy dysfunctions could explain the diverse pathological course of the disorders. Analyzing in more detail these autophagy mediators and the molecular link to the specific disease genes, AD and FTD-related mediator proteins appears to be mainly associated to the initiation complex of the macroautophagy cascade, involving mainly beclin 1 interactome: B-cell CLL/lymphoma 2 (BCL2), BCL2-like 1 (BCL2L1), and Atg14 (Figure 6). Beclin 1 interactome contains stimulating and suppressive components regulating the initiation of the autophagosome formation and, recently, Beclin 1 has been found to be down-regulated in AD brain. Moreover, suppression of Beclin 1 in cultured neurons and transgenic mice induces the deposition of amyloid-β peptides, whereas its overexpression reduces its accumulation [42]. Beclin 1 protein also assembles two core complexes, Atg14L or UVRAG complexes, and with Atg14L protein induces the phagophore formation and, thus, stimulates autophagocytosis, whereas the UVRAG/Beclin 1 complex controls other Beclin 1-dependent functions, e.g. phagocytosis. The subcellular compartmentation of Beclin 1 is regulated by different molecules including BCL-2 [43] and mTOR [44] which are represented in the mediator list. Several proteins can control the activation of beclin complex including two protein kinases included in our mediator list: CKD2 and CDK5 [45] (Additional file 1: Table S1). Atg5 protein, a mediator related to both AD and FTD disease genes (Figures 5 and 6), is also essential for the autophagy process and, in a conjugated form with Atg12 and Atg8 (LC3), is involved in the early stages of autophagosome formation (Figure 7C). Taken together these results suggest impairment in the early stages of autophagy complex essential for autophagosome formation [46,47], including protein kinase network regulating autophagosome assembling. The hypothesis that autophagy regulation and, in particular, its induction could contribute to AD pathology is also supported by recent evidence suggesting that the synthesis of components of the lysosome is up-regulated at the transcriptional and translational levels in the AD brain and AD mouse models [48-54]. Moreover, Lipinski et al. [55] recently reported an up-regulation of the transcription of genes stimulating autophagy and a down-regulation of the negative regulators of autophagy in the brains of AD affected subjects. In the dementia network, this interactome is connected to presenilin (Figure 5) whose mutation underlies the majority of familial Alzheimer’s disease cases [56-58] and whose role in autophagy has been shown to be central [59], presenilin 1 being essential for lysosome acidification, and proteolysis during autophagy [60].

Frontotemporal dementia-related mediator proteins seem to be involved not only in the vesicle elongation and autophagosome assembling process, but also, and exclusively, to vesicle nucleation procedure (Figure 7C). This process includes WIPI proteins (WD-repeat protein interacting with phosphoinosides), WIPI-1 and WIPI-2, evolved from the yeast ancestral autophagy protein Atg18 (Proikas-Cezanne T, 2004; Polson HE, 2010) as membrane components of autophagosomes (Mauthe 2011, [61]). Both WIPI-1 and WIPI-2 specifically bind PtdIns(3)P and localize at autophagosomal membranes (phagophore) [62].

TARDPB (TDP-43) appears to be a central protein in our autophagy-related sub-network (Figure 5). TDP-43 is a DNA/RNA-binding protein with multicellular functions. Several mutations of its gene have been reported in cases of frontotemporal lobar degeneration (FTLD) [63]. It is processed and degraded by both autophagy and the ubiquitin-proteasome systems [64]. Activation of autophagy by rapamycin plays an active role in the clearance of TDP-43 deficits in mouse model with proteinopathies of the TAR DNA-binding protein 43 [65]. Depletion of TDP-43 induces a down-regulation of the major autophagy component Atg7, causes impairment of autophagy and facilitates the accumulation of polyubiquitinated proteins which could be rescued by overexpression of the protein, with a feedback regulatory loop between TDP-43 and autophagy [64]. In our network, TDP-43 is linked to AMPK subunit PRKAA2 and a functional link between these two proteins has been suggested in pathological conditions showing that activated AMPK adversely affects mutant TDP-43-induced motor neurons diseases [66]. In addition, it is related to other central autophagy proteins such as ATG5 and ATG16L, which create a multimeric complex playing an essential role in autophagosome formation, a system highly conserved in all eukaryotes [67]. The other proteins linked to TDP-43 are WIPI1 and 2 (Figure 5). Thus, these findings could suggest a therapeutic modulation of autophagy involving approaches that functionally target WIPI proteins and ATG5-ATG16 complex for the treatment of FTD and other diseases involving mutations in TDP-43 gene.

Apart from the metabolic-associated biological processes terms, cell surface receptor signaling pathway-related terms were also highly significant enriched, in particular in proteins associated to the Wnt pathway (Figures 3 and 4). Several proteins in the mediator list are represented in the pathway (see Additional file 4: Figure S4) including GSK-3β, a tau kinase that was also included in the most connected mediator proteins list (Table 3) and in the autophagy-related proteins (Additional file 3: Table S3). Several preclinical and clinical data strongly link GSK-3β to dementia: different inhibitors of *GSK3B* activity block neurodegeneration *in vitro*, and GSK-3β -mediated Wnt signaling can mediate amyloid peptide toxicity in vitro [68,69]. Finally, in human postmortem brain, this protein is physically associated with neurofibrillary tangles, one of the pathologic hallmarks of AD [70]. WNT pathway has also been recently linked to autophagy. In fact, autophagy negatively regulates Wnt signalling by promoting Dishevelled (Dvl) degradation, with a role for Von Hippel–Lindau protein-mediated ubiquitylation [71], both of them present in the dementia network mediator list.

In our dementia network, among the drug targets associated to the autophagy-related mediators, the highest represented proteins are subunits of the Protein phosphatase 2A (PP2A; Figure 5), a serine/threonine-specific protein phosphatase consisting of A, B and C subunits that plays multiple roles in different signaling pathways and regulates diverse cellular processes. Among the six PP2A proteins, three proteins (PPP2R2B, PPP2CA, and PPP2R1A) are also listed in the highly ranked proteins in the dementia network (Table 3), demonstrating their centrality. A recent study confirms that PP2A blockade inhibits autophagy potentially through activation of AMPK [72]. A role of PP2A in dementia is further demonstrated by the evidence that okadaic acid and calyculin A, two potent PP2A inhibitors [73], are able to induce tauopathy and cognitive deficiency in rats [74,75]. Thus, PP2A subunits could be considered as a potential therapeutic target for AD.

In our drug targets list related to autophagy mediators (Figure 6), other molecular targets could be considered suitable for therapeutic intervention including AMPK-related proteins, a highly ranked protein in our network (Table 3 and Additional file 3: Table S3) and a target which has been already considered for the treatment of Alzheimer’s disease. In fact, pioglitazone, an antidiabetic drug which acts also by activating AMPK [76], has been proven to reverse pathological conditions in an animal model of the disease [77] and it is in clinical trial for Alzheimer’s disease (http://www.clincaltrial.gov).

In more general terms, a direct action on the regulation of autophagy, potentially an activation of the autophagic process should be considered to the development of optimal therapeutics, although autophagy can function both as a cytoprotective mechanism, but it also has the capacity to cause cell death.

## Conclusion

This network analysis considering the established knowledge on different neurodegenerative dementia disease represented by OMIM data and the drug targets in the different phases of the drug discovery process, identifies the autophagy process as a central dis-regulated pathway in these sub-group of neurodegenerative disorders. We could hypothesize that different mutation or alteration at the genomic level could affect different phases of the autophagy process and thus therapeutic modulation could involve approaches that functionally target the specific proteins. Exploring the molecular mechanisms of autophagy opens an avenue for development of novel drugs and particularly, these results could suggest the potentiality of drug targeting specific PP2A subunits for the treatment of dementia.

## Abbreviations

FTD: Frontotemporal dementia; AD: Alzheimer disease; LBD: Lewy bodies disease; PSP: Progressive supranuclear palsy; CBD: Corticobasal dementia; HD: Hungtington’s disease; ALSPD: Amyotrophic lateral sclerosis-Parkinsonism/dementia complex; PIN: Protein-protein interaction network; GSK-3β: Glycogen synthase kinase beta; AMPK: AMP-regulated kinase; TDP-43: TAR DNA-binding protein 43; PP2A: Protein phosphatase 2A.

## Competing interests

The authors declare that there is no competing interest in relation to the publication of this article.

## Authors’ contributions

LC and TPN conceived and designed the study, collected and analyzed the data and wrote the paper. Both authors read and approved the final manuscript.

## Supplementary Material

Additional file 1: Table S1 List of Drug targets, list of mediator proteins in the dementia network, and gene ontology biological functions enrichment analysis results of all mediators, mediator related to tauopathies and highly ranked protein in the dementia network. Click here for file

Additional file 2: Figure S2 Summary of statistically significant Gene Ontology biological processes functional annotation corresponding to proteins in the highly ranked proteins as obtained from REVIGO [28]. Nodes are GO terms and edges represent the strongest GO terms pairwise similarity. Colors represent the p-values (low values in green, high in red). Only significant GO terms are shown (P < 0.001). Click here for file

Additional file 3: Table S3 List of 45 mediators (with their degree centrality related to dementia and autophagy [29] networks) found in the autophagy interaction network [29]. In bold are proteins that play a role as the articulation points in the human autophagy network. Click here for file

Additional file 4: Figure S4 Schematic Figure representing the WNT pathway as described in the Biocarta database. In red are labeled the dementia network mediator proteins. Click here for file
