# Supplementary figures and images for: A systems biology investigation of neurodegenerative dementia reveals a pivotal role of autophagy
Source: BMC Syst Biol. 2014 Jun 7;8:65. doi: 10.1186/1752-0509-8-65 (PMC4077228; doi:10.1186/1752-0509-8-65)

## Slide 1
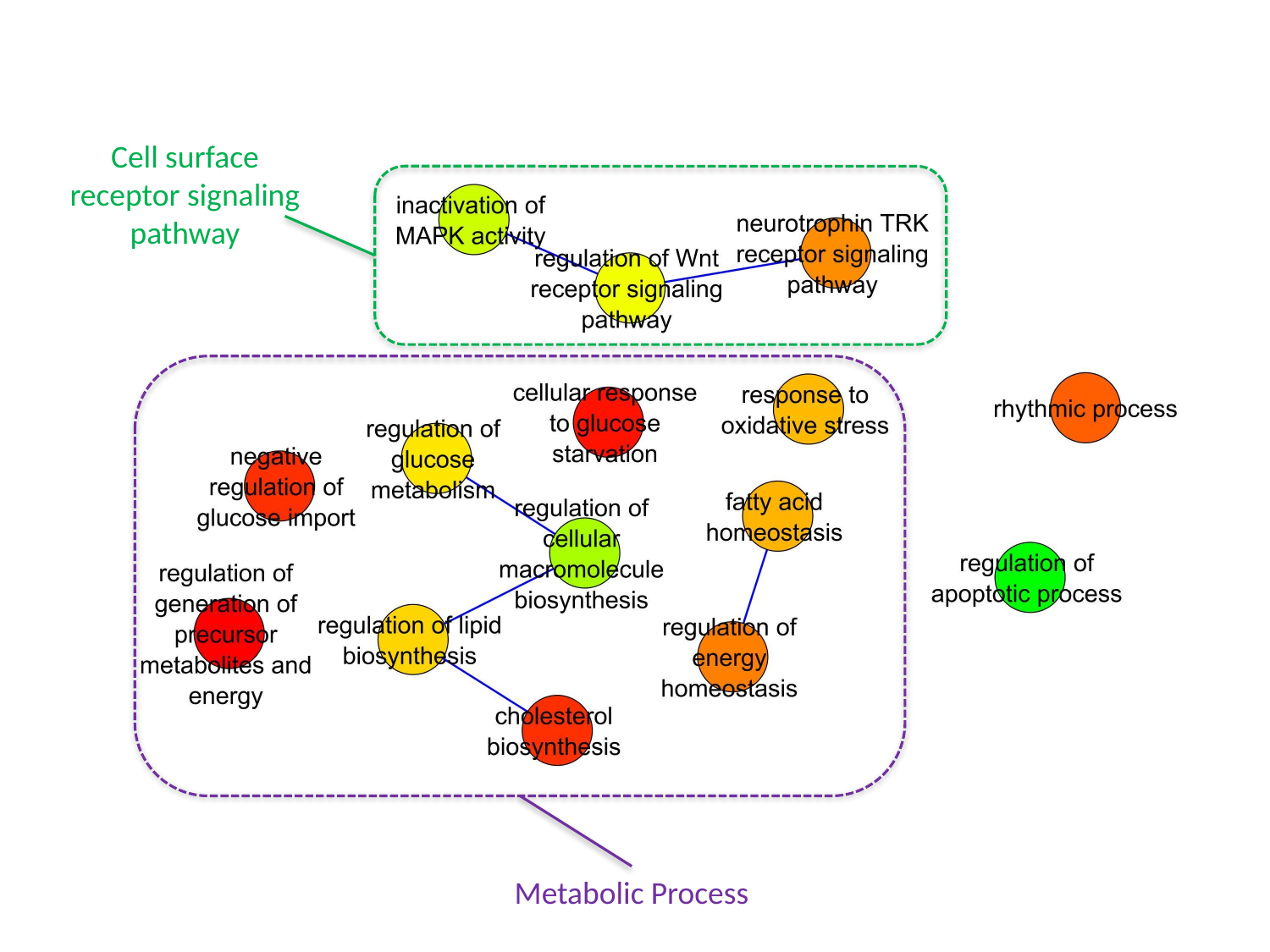

Cell surface receptor signaling pathway
Metabolic Process

Supplement: Additional file 2: Figure S2 — Summary of statistically significant Gene Ontology biological processes functional annotation corresponding to proteins in the highly ranked proteins as obtained from REVIGO [28]. Nodes are GO terms and edges represent the strongest GO terms pairwise similarity. Colors represent the p-values (low values in green, high in red). Only significant GO terms are shown (P < 0.001). [file 1752-0509-8-65-S2.pptx]

## Slide 1
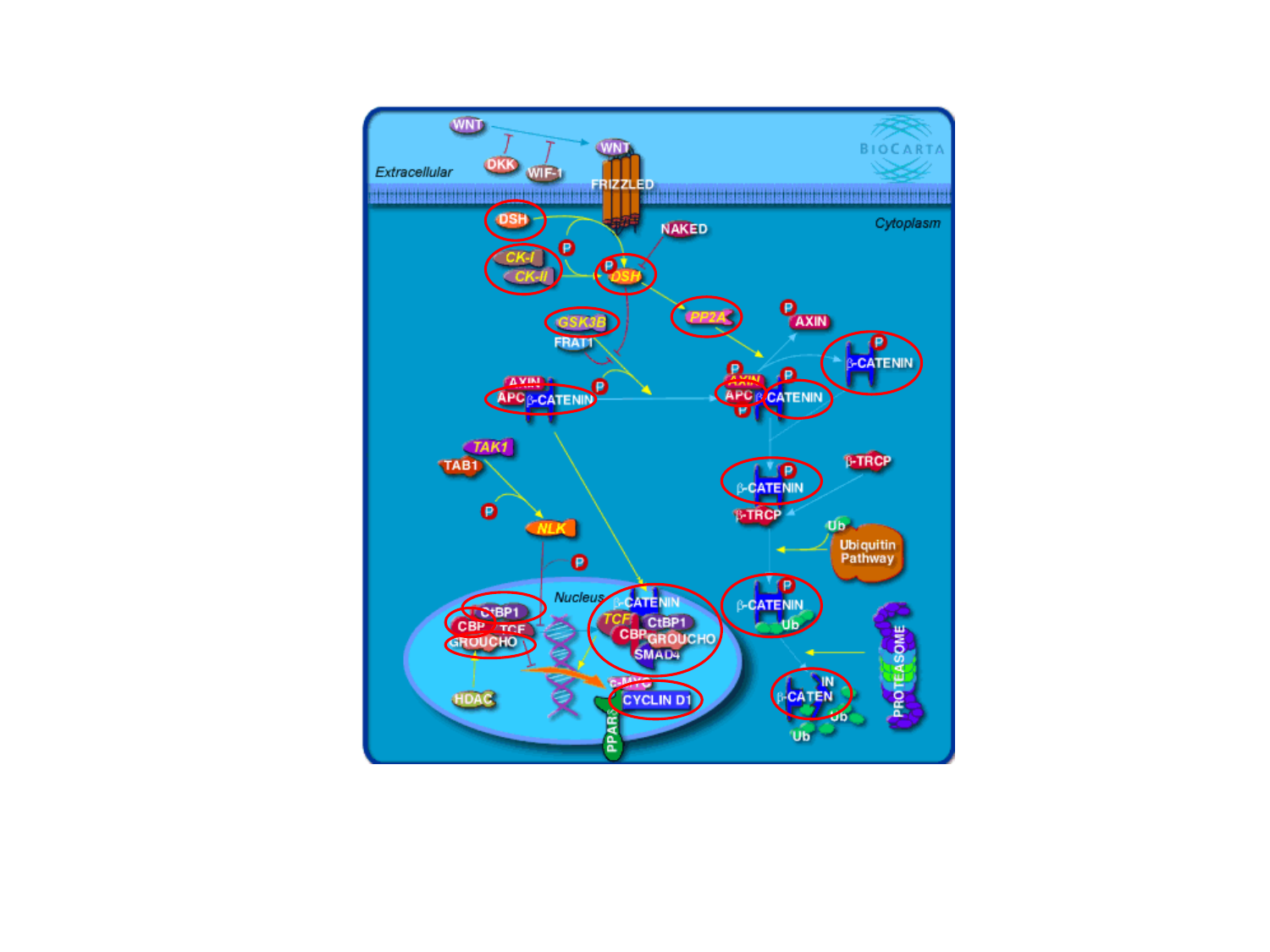

Supplement: Additional file 4: Figure S4 — Schematic Figure representing the WNT pathway as described in the Biocarta database. In red are labeled the dementia network mediator proteins. [file 1752-0509-8-65-S4.pptx]
