# Supplementary material for: A systems biology investigation of neurodegenerative dementia reveals a pivotal role of autophagy
Source: BMC Syst Biol. 2014 Jun 7;8:65. doi: 10.1186/1752-0509-8-65 (PMC4077228; doi:10.1186/1752-0509-8-65)
Supplement: Additional file 3: Table S3 — List of 45 mediators (with their degree centrality related to dementia and autophagy [29] networks) found in the autophagy interaction network [29]. In bold are proteins that play a role as the articulation points in the human autophagy network. [file 1752-0509-8-65-S3.docx]

| **Gene Name** | **Degree Centrality in Dementia Network** | **Degree Centrality in Autophagy Network** |
| --- | --- | --- |
| **PRKAA1** | 419 | 16 |
| **PRKAA2** | 392 | 30 |
| GSK3B | 170 | 1 |
| CALM1 | 19 | 1 |
| **SQSTM1** | 18 | 29 |
| **GBAS** | 12 | 28 |
| **UVRAG** | 11 | 8 |
| YWHAH | 11 | 1 |
| YWHAE | 11 | 1 |
| **CAMKK2** | 10 | 18 |
| ATG5 | 10 | 8 |
| **CLN3** | 9 | 42 |
| **KBTBD7** | 9 | 27 |
| **WIPI2** | 9 | 7 |
| **NEK9** | 9 | 6 |
| FLNA | 9 | 1 |
| CLTC | 9 | 1 |
| **NSF** | 8 | 15 |
| **DDA1** | 7 | 42 |
| PRKAG1 | 7 | 12 |
| **TRAF2** | 7 | 8 |
| YWHAB | 7 | 1 |
| **C12ORF44** | 6 | 32 |
| **GABARAP** | 6 | 23 |
| PRKAG2 | 6 | 16 |
| **ATG16L1** | 5 | 11 |
| **TECPR1** | 5 | 9 |
| **STK11** | 5 | 6 |
| WDR45 | 5 | 3 |
| MAP1B | 4 | 9 |
| **NRBF2** | 4 | 6 |
| **AMBRA1** | 4 | 5 |
| KIAA0831 | 4 | 4 |
| SH3GLB1 | 4 | 4 |
| **GABARAPL1** | 3 | 32 |
| **MAP1LC3C** | 3 | 12 |
| PIK3C2A | 3 | 9 |
| **WIPI1** | 3 | 4 |
| MCM2 | 3 | 3 |
| **DDIT3** | 3 | 3 |
| CTNND1 | 3 | 1 |
| MAP3K5 | 2 | 2 |
| PSMC2 | 2 | 1 |
| TP53BP2 | 2 | 1 |
| PFKL | 2 | 1 |
